# SERPINA3K Prevents Oxidative Stress Induced Necrotic Cell Death by Inhibiting Calcium Overload

**DOI:** 10.1371/journal.pone.0004077

**Published:** 2008-12-30

**Authors:** Bin Zhang, Jian-xing Ma

**Affiliations:** Department of Cell Biology, Department of Medicine, The University of Oklahoma Health Sciences Center, Oklahoma City, Oklahoma, United States of America; National Institutes of Health, United States of America

## Abstract

**Background:**

SERPINA3K, an extracellular serine proteinase inhibitor (serpin), has been shown to have decreased levels in the retinas of diabetic rats, which may contribute to diabetic retinopathy. The function of SERPINA3K in the retina has not been investigated.

**Methodology/Principal Findings:**

The present study identified a novel function of SERPINA3K, i.e. it protects retinal cells against oxidative stress-induced cell death including retinal neuronal cells and Müller cells. Flow-cytometry showed that the protective effect of SERPINA3K on Müller cells is via reducing oxidation-induced necrosis. Measurements of intracellular calcium concentration showed that SERPINA3K prevented the intracellular calcium overload induced by H_2_O_2_. A similar protective effect was observed using a calcium chelator (BAPTA/AM). Further, SERPINA3K inhibited the phosphorylation of phospholipase C (PLC)-gamma1 induced by H_2_O_2_. Likewise, a specific PLC inhibitor showed similar protective effects on Müller cells exposed to H_2_O_2_. Furthermore, the protective effect of SERPINA3K was attenuated by a specific PLC activator (m-3M3FBS). Finally, in a binding assay, SERPINA3K displayed saturable and specific binding on Müller cells.

**Conclusion/Significance:**

These results for the first time demonstrate that SERPINA3K is an endogenous serpin which protects cells from oxidative stress-induced cells death, and its protective effect is via blocking the calcium overload through the PLC pathway. The decreased retinal levels of SERPINA3K may represent a new pathogenic mechanism for the retinal Müller cell dysfunction and neuron loss in diabetes.

## Introduction

The serpin super-family consists of intracellular and extracellular serpins, based on their locations [1]. Previous studies have suggested that the extracelluar and intracellular serpins have different functions and mechanisms of action [1], [2]. A recent study has shown that an intracellular serpin inhibits cell necrosis induced by hypoxia or oxidation [3]. However, the role of extracellular serpins in cell necrosis has not been reported [4].

SERPINA3K was first identified as a specific inhibitor of tissue kallikrein and thus named kallikrein-binding protein [5], [6]. Amino acid sequence analysis classified SERPINA3K into the serine proteinase inhibitor (serpin) family [1]. Tissue kallikrein is a serine proteinase and releases bioactive kinins from kininogens [7], [8]. The kallikrein-kinin system has important functions in inflammation, blood pressure regulation, generating pain and allergy [9]. SERPINA3K specifically binds to tissue kallikrein, forming a covalent complex and inhibits proteolytic activities of tissue kallikrein [6]. SERPINA3K participates in the regulation of vasodilation and local blood flow via interactions with the kallikrein-kinin system [10]. Later studies suggest that SERPINA3K has other functions independent of inhibition of tissue kallikrein. SERPINA3K has been found to inhibit angiogenesis and to reduce vascular permeability [11], [12]. These effects of SERPINA3K have been shown to be independent of its interactions with the kallikrein-kinin system [11].

SERPINA3K is expressed at high levels in the liver, and lower levels in other tissues, such as the kidney, pancreas and retina. SERPINA3K levels have been shown to decrease in the retina of a diabetic rat model, suggesting that decreased SERPINA3K levels may contribute to diabetic retinopathy [13].

Retinal Müller cells are principal glial cells in the retina and in contact with every type of neuronal cells in the retina. Retinal Müller cells play important roles in supporting the retinal neurons and in neuronal signal processing [14]–[16]. Müller cell death has been found to lead to photoreceptor apoptosis and retinal degeneration [17]. The oxidation-induced Müller cell dysfunction has been implicated in diabetic retinopathy [18]. Therefore, Müller cells are commonly used as a model for studying neuroprotective factors and retinal degeneration.

Oxidative stress is believed to play an important pathogenic role in diabetic retinopathy [19]. It induces retinal neuron degeneration as well as inflammation and vascular injury. Reactive oxygen species (ROS) such as superoxide, a highly reactive hydroxyl radical, and hydrogen peroxide (H_2_O_2_) are physiological mediators of cellular responses [20]. Increased ROS generation in the retina is a characteristic of the oxidative stress as found in diabetic retinopathy [21]. H_2_O_2_ is commonly used as a stressor to induce oxidative stress in experimental models. Exogenous H_2_O_2_ is known to lead to multiple cellular responses, such as kinase activation, ion overload, etc. H_2_O_2_ is also a stimulator in the apoptotic and necrotic pathways [22], [23].

In the present study, we used the exogenous H_2_O_2_ as an oxidative stressor to investigate the protective role of SERPINA3K in cultured retinal cells and explored its mechanism of action.

## Results

### A novel protective effect of SERPINA3K

**Figure 1:**
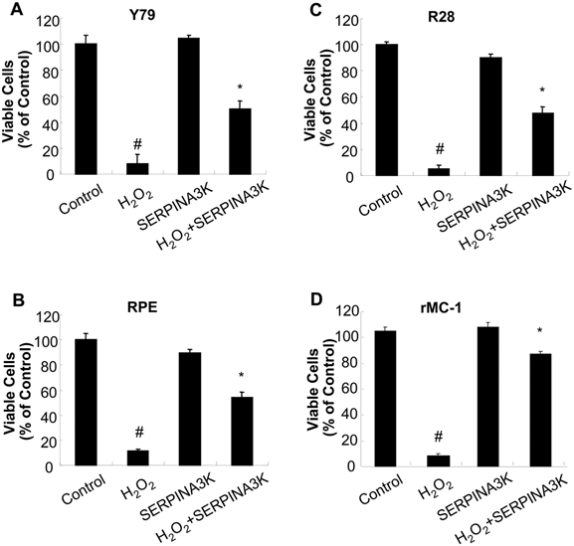
Protective effects of SERPINA3K in retinal cells. Y79 (A), R28 (B), RPE (C) and rMC-1 cells (D) were exposed to 400 µM H_2_O_2_ in the presence of 1 µM SERPINA3K or BSA for 8 h, or incubated with 1 µM SERPINA3K alone for 8 h. Viable cells were quantified by MTT assay (mean±SEM, n = 3). * P<0.01, versus the H_2_O_2_ treated cells. # P<0.01, versus the control cells.

To investigate if SERPINA3K protects retinal neurons under oxidative stress, we established an *in vitro* model of H_2_O_2_-induced cell death, as H_2_O_2_ is a highly membrane permeable oxidant [24] and plays important roles in both apoptosis [22] and necrosis [23] under oxidative stress. We have compared the effects of SERPINA3K on cell death induced by H_2_O_2_ in Y79, a retinoblastoma cell line, R28, a rat retinal precursor cell line, RPE cells and rMC-1, a cell line derived from rat retinal Müller cells. As shown by MTT assay, exposure to H_2_O_2_ (400 µM) for 8 h resulted in significant cell death in all of the cell lines. Treatment with 1 µM SERPINA3K significantly decreased cell death in Y79, R28, RPE and rMC-1 cells exposed to H_2_O_2_, by 40%, 44%, 42% and 81%, respectively, when compared to the treatment with BSA at the same concentration (Fig. 1). At the same concentration, however, SERPINA3K displayed different levels of the protective effects on different cell lines.

### SERPINA3K protects cells against H_2_O_2_-induced cell death

**Figure 2:**
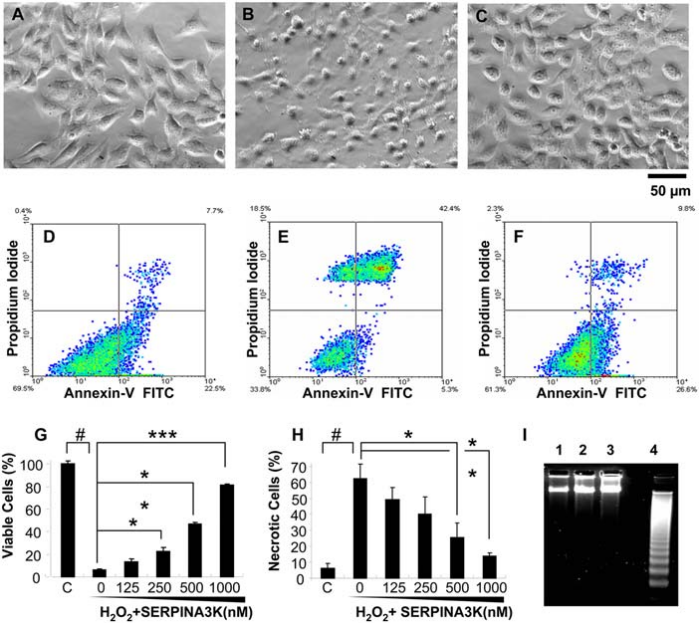
Protective effects of SERPINA3K on rMC-1 cells against H_2_O_2_-induced necrosis. Müller-derived rMC-1 cells were pre-treated with SERPINA3K or BSA for 1 h and then exposed to 400 µM H_2_O_2_ for 8 h (A–C, G) and 4 h (D–F, H, I). The protein concentration in the media was brought to 1 µM in each well by addition of BSA. (A–C) Representative phase contrast images showing cell morphology in untreated control (A), cells treated with 1 µM BSA and H_2_O_2_ (B), and cells treated with 1 µM SERPINA3K and H_2_O_2_ (C). Scale bar, 50 µm. (G) Viable cells were quantified using the MTT assay (mean±SEM, n = 3). (D–F, H) The viable and necrotic cells were analyzed by flow cytometry following staining with PI and Annexin-V; (D) Untreated control; (E) treated with 1 µM BSA and H_2_O_2_; (F) treated with 1 µM SERPINA3K and H_2_O_2_; (H) quantification of necrotic cells as percentages of total cells (mean±SEM, n = 3). (I) DNA laddering analysis showed no apparent DNA fragmentation in the H_2_O_2_-treated cells. Lane 1, control cells; 2, cells treated with H_2_O_2_ for 4 h; 3, cells treated with H_2_O_2_ and SERPINA3K, and 4, over-grown cells as positive control. # P<0.001, H_2_O_2_ treated cells versus control cells. * P<0.05, ** P<0.01, *** P<0.001, the cells treated by different doses of SERPINA3K versus the cells treated only by BSA.

Retinal Müller-derived rMC-1 cells were chosen for the further mechanism study, as Müller cells in the retina are essential for the survival of the retinal neuronal cells, and rMC-1 also displayed the highest response for SERPINA3K. To evaluate the cell protective effect of SERPINA3K under oxidative stress, the rMC-1 cells were exposed to 400 µM H_2_O_2_ for 8 h in a culture medium containing 1% FBS. In the cells without the SERPINA3K treatment, the H_2_O_2_ exposure induced apparent morphological changes in most of the cells, exhibiting nuclear swelling while still attaching to the bottom of the well (Fig. 2B), a morphological change of necrotic cells. The 1-h pre-treatment with SERPINA3K resulted in an apparent improvement of cell morphology (Fig. 2C).

Quantification of viable cells by MTT assay showed that H_2_O_2_ induced significant decreases in cell viability, while SERPINA3K, in a range of 125–1000 nM, showed a dose-dependent protection of Müller-derived rMC-1 cells from H_2_O_2_–induced cell death (Fig. 2G).

### SERPINA3K prevents necrosis induced by H_2_O_2_

The retinal Müller-derived rMC-1 cells exposed to H_2_O_2_ were stained with PI/Annexin-V, and necrotic and apoptotic cells were quantified by flow cytometry. Histogram of flow cytometry showed increased numbers of the PI-positive cells, with the broken cell membrane, in the H_2_O_2_-treated group. However, there was no significant increase of cell number in the lower-right quadrant of the histogram, which represents the Annexin-V-positive cells or apoptotic cells (Fig. 2E). This result suggests that the cell death induced by acute exposure to H_2_O_2_ is mostly through the necrotic rather than the apoptotic pathway. DNA fragmentation analysis was used to distinguish between apoptosis and necrosis. The DNA fragmentation induced by cell over-growth was used as the positive control of apoptotic cells (Fig. 2I). In the sub-confluent rMC-1 cells exposed to 400 µM H_2_O_2_ for 4 h, no fragmented DNA was detected by gel electrophoresis (Fig. 2I), further suggesting that the cell death induced by acute H_2_O_2_ exposure is through necrosis.

To quantify the effect of SERPINA3K on necrosis induced by H_2_O_2_, the PI-stained necrotic cells were counted by flow cytometry. As shown in Figure 2H, 400 µM H_2_O_2_ exposure generated 61% necrotic cells, compared to 8% in the untreated control cells (Fig. 2D,E). Pre-treatment of the cells with 1 µM SERPINA3K prior to the H_2_O_2_ exposure resulted in a significant decrease of necrotic cells (12% necrotic cells compared to 61% in the BSA control, p<0.001, n = 3) (Fig. 2F). Moreover, the protective effect of SERPINA3K appears to be concentration-dependent (Fig. 2H).

### The protective effect of SERPINA3K is not through blocking ROS generation

**Figure 3:**
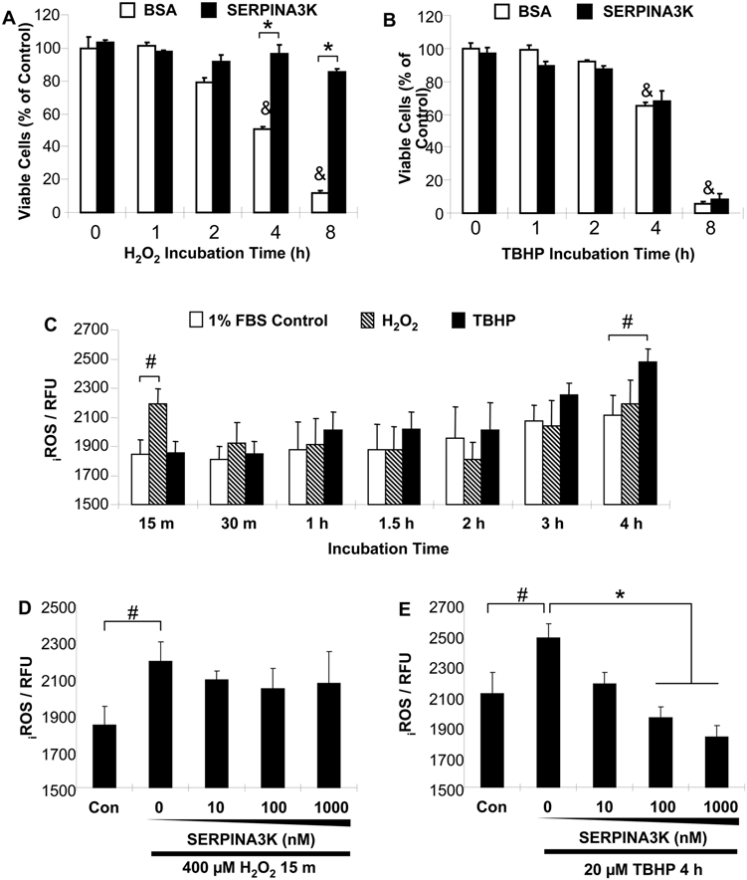
Lack of effect of SERPINA3K on the intracellular ROS generation in cells exposed to H_2_O_2_. (A, B) The rMC-1 cells were treated with 400 µM H_2_O_2_ (A) or 20 µM TBHP (B) for 1, 2, 4 and 8 h in the absence or presence of 1 µM BSA or SERPINA3K. Viable cells were quantified using MTT assay (mean±SEM, n = 3). (C) The rMC-1 cells were exposed to 400 µM H_2_O_2_ or 20 µM TBHP for various durations as indicated, to define the time point for the ROS increase. Note that ROS levels were elevated by H_2_O_2_ as early as 15 min, while the ROS generation induced by TBHP occurred at 4 h of the treatment. (D&E) rMC-1 cells were pre-treated with various concentrations of SERPINA3K for 1 h. The medium was supplemented with BSA to the same total protein concentration in each well. The cells were then exposed to 400 µM H_2_O_2_ for 15 min (D) or to 20 µM TBHP for 4 h (E). The intracellular ROS generation was measured using CM-H2DCFDA as a probe. Values are arbitrary fluorescence units (mean±SEM, n = 8). SERPINA3K blocked the ROS increase induced by TBHP but not that by H_2_O_2_. * P<0.01, the SERPINA3K-treated cells versus the BSA-treated cells. & P<0.01, the BSA-treated cells exposed to H_2_O_2_ versus the BSA-treated cells without H_2_O_2_ exposure. # P<0.05, the H_2_O_2_ or TBHP-treated cells versus control cells.

To determine if SERPINA3K protects Müller-derived rMC-1 cells against the injury induced by different types of oxidative stressors, we have employed Tert-butyl hydroperoxide (TBHP), which is known to induce lipid peroxidation and ROS formation. Müller-derived rMC-1 cells were treated with 400 µM H_2_O_2_ or 20 µM TBHP for 1, 2, 4 and 8 h in a medium containing 1% FBS. In the absence of SERPINA3K (with 1 µM BSA as control), H_2_O_2_ and TBHP both induced the exposure time-dependent cell death, up to 90% after an 8-h incubation (Fig. 3A, B). SERPINA3K significantly decreased the cell death induced by H_2_O_2,_ but not that induced by TBHP (Fig. 3A, B).

Oxidative stress is known to induce the intracellular ROS generation, which can activate the apoptotic pathway or necrotic pathway. Here we investigated whether SERPINA3K prevents necrosis in Müller-derived rMC-1 cells via decreasing intracellular ROS levels in cells exposed to H_2_O_2_, using a fluorometric microplate assay with CM-H2DCFDA as the probe. H_2_O_2_ treatment induced a marked increase of intracellular ROS levels after a 15-min exposure but not at later time points, compared to the control. Since H_2_O_2_ is a highly membrane permeable oxidant, the intracellular ROS detected at 15 min of H_2_O_2_ treatment could be originated from penetration of extracellular H_2_O_2_. SERPINA3K did not affect ROS levels in the cells exposed to H_2_O_2_ for 15 min (Fig. 3D). In contrast, the TBHP treatment showed a time-dependent increase of intracellular ROS (Fig. 3C). SERPINA3K reduced the TBHP-induced intracellular ROS generation in a dose-dependent manner (Fig. 3E), but it did not protect the cells from TBHP-induced cell death (Fig. 3B). This result suggests that the effect of SERPINA3K on H_2_O_2_-induced cell death is not related to decrease of ROS levels.

### The protective effect of SERPINA3K is through blocking intracellular Ca^2+^ overload induced by H_2_O_2_

**Figure 4:**
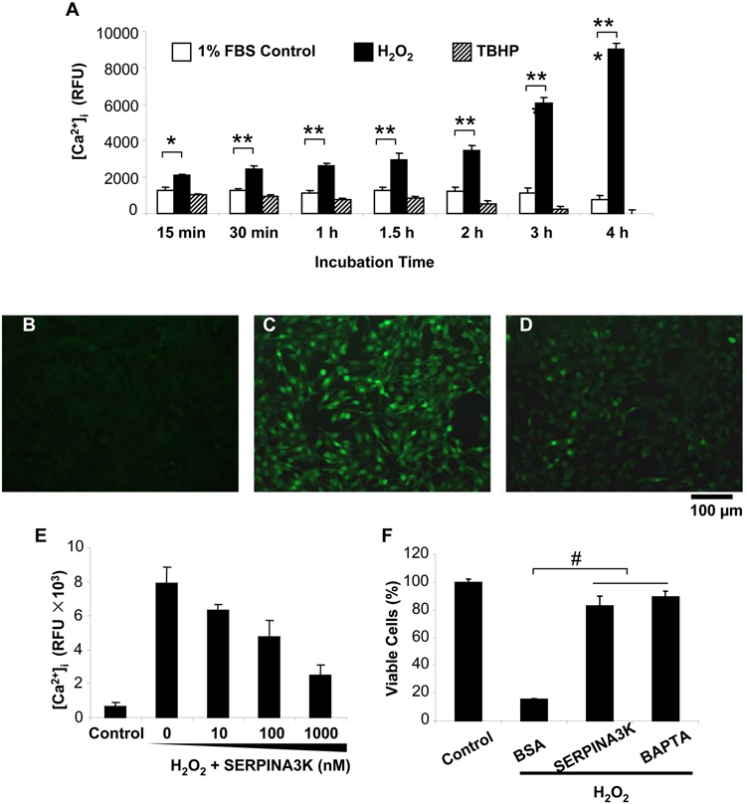
SERPINA3K blocks intracellular calcium overload induced by H_2_O_2_. (A) Elevated intracellular [Ca^2+^] in cells exposed to H_2_O_2_. The Müller-derived rMC-1 cells were treated with 400 µM H_2_O_2_ for various durations as indicated. TBHP (20 µM) was used as control. Intracellular [Ca^2+^] was measured by the fluorescence of the probe Fluo-4/AM (mean±SEM, n = 8). (B–E) The cells were pre-treated with 1 µM SERPINA3K or BSA for 1 h followed by the H_2_O_2_ exposure. BSA was added to bring the total protein concentration to the same in each well. Representative fluorescence images were captured under a fluorescent microscope from untreated cells (B), cells pre-treated with 1 µM BSA (C) and with 1 µM SERPINA3K (D) followed by a 3-h exposure to H_2_O_2_. (E) [Ca^2+^] in cells pre-treated with various concentrations of SERPINA3K prior to the exposure to H_2_O_2_ (mean±SEM, n = 6). (F) The protective effects of 1 µM SERPINA3K and 10 µM BAPTA/AM (a calcium chelator) were quantified using the MTT assay (mean±SEM, n = 3). * P<0.05, ** P<0.01, *** P<0.001, the cells treated by H_2_O_2_ versus the control cells. # P<0.01, the SERPINA3K or BAPTA-treated cells versus the BSA-treated cells. Scale bar, 100 µm.

The calcium-induced protease release has been shown to be one of the major inducers of necrosis [25], [26]. To determine the intracellular Ca^2+^ concentration in Müller-derived rMC-1 cells under the oxidative stress, the fluorescence of the probe Fluo-4/AM, which reacts with intracellular free Ca^2+^ to generate a green fluorescence, was measured. H_2_O_2_ induced a time-dependent Ca^2+^ overload in rMC-1 cells (Fig. 4A), while TBHP did not increase the intracellular Ca^2+^ concentration, although it induces ROS generation (Fig. 4A).

The control rMC-1 cells in the medium with 1% FBS showed only basal levels of green fluorescence, indicating a low intracellular Ca^2+^ concentration (Fig. 4B). H_2_O_2_ exposure for 3 h substantially increased intracellular Ca^2+^ levels (Fig. 4C). Pre-treatment with SERPINA3K prevented the intracellular Ca^2+^ overload induced by H_2_O_2_ (Fig. 4D) in a dose-dependent manner (Fig. 4E).

We have also measured the protective effect of BAPTA/AM, a calcium chelator, on Müller-derived rMC-1 cells. At 10 µM, BAPTA/AM reduced intracellular Ca^2+^ concentrations and prevented rMC-1 cell death induced by H_2_O_2_, similar to that of 1 µM SERPINA3K (Fig. 4F), suggesting that the protective effect of SERPINA3K is through blockade of the calcium overload induced by H_2_O_2_.

### SERPINA3K does not chelate free Ca^2+^

**Figure 5:**
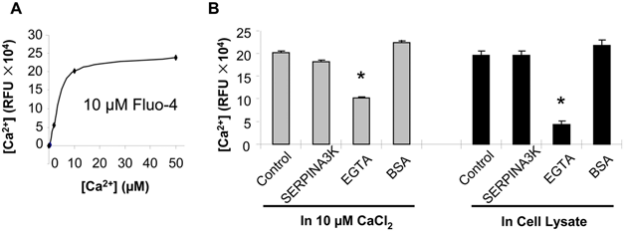
SERPINA3K does not chelate free calcium. Free [Ca^2+^] was measured by the fluorescence of the Fluo-4 (pentapotassium salt). (A) A Ca^2+^/Fluo-4 binding curve was plotted with different concentrations of CaCl_2_ and 10 µM Fluo-4. (B) SERPINA3K (10 µM) was incubated with 10 µM CaCl_2_ or a cell lysate in the presence of 10 µM Fluo-4. EGTA (10 µM) and BSA (10 µM) were used as positive and negative controls, respectively. Free [Ca^2+^] was measured (mean±SEM, n = 3). * P<0.01, versus control cells.

To determine whether SERPINA3K prevents calcium overload through chelating Ca^2+^, an *in vitro* Ca^2+^ binding assay was performed by measuring the fluorescence of the calcium-bound Fluo-4 (pentapotassium salt). In a 96-well plate, each well contained 100 µl 10 µM Fluo-4 and 2, 10 and 50 µM CaCl_2_. As shown by the binding curve, 10 µM CaCl_2_ reached the saturation level of the fluorescence when 10 µM Fluo-4 was used (Fig. 5A). SERPINA3K was added to the well to a final concentration of 10 µM, with 10 µM EGTA and BSA as positive and negative controls. The Ca^2+^ fluorescence was decreased by EGTA, but not by SERPINA3K (Fig. 5B), suggesting that SERPINA3K did not chelate free Ca^2+^.

We also measured potential calcium chelating activity of SERPINA3K in a cell lysate containing Ca^2+^. The cell lysate was diluted with PBS to generate the same fluorescence intensity as that of 10 µM CaCl_2_ in 10 µM Fluo-4. In the cell lysate, 10 µM SERPINA3K did not decrease the fluorescence of Fluo-4 (Fig. 5B, right panel), suggesting that SERPINA3K inhibited the calcium overload via a mechanism rather than chelating the free calcium ion.

### SERPINS3K blocks the H_2_O_2_–induced calcium overload via the PLC pathway

**Figure 6:**
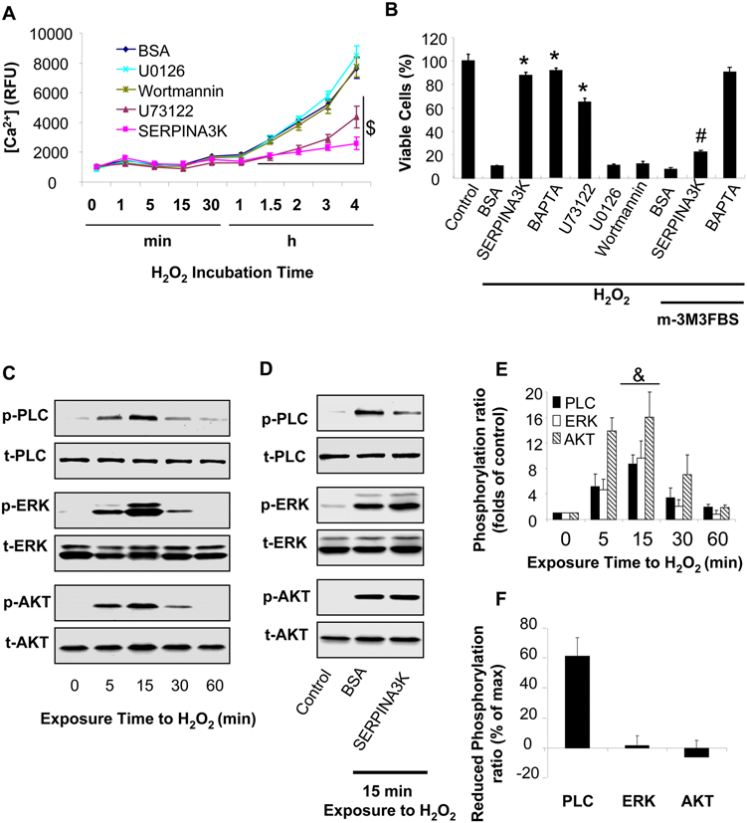
SERPINA3K inhibits the H_2_O_2_-induced calcium overload and necrosis via PLC but not the ERK or PI3K/Akt pathways. (A) The rMC-1 cells were treated with 400 µM H_2_O_2_ in the presence of 1 µM SERPINA3K, 250 nM U73122 (PLC inhibitor), 10 µM U0126 (ERK inhibitor), 100 nM Wortmannin (PI3K inhibitor) or 10 µM BAPTA. BSA (1 µM) was used as negative control. Intracellular [Ca^2+^] was measured using the fluorescence of the probe Fluo-4/AM at different intervals of the treatment (mean±SEM, n = 6). (B) The protective effects of the inhibitors and 1 µM SERPINA3K against the H_2_O_2_-induced cell death with or without 10 µM m-3M3FBS (PLC activator) were quantified by the MTT assay after exposure to H_2_O_2_ for 8 h (mean±SEM, n = 3). (C) The rMC-1 cells were treated with 400 µM H_2_O_2_. The cells were harvested at different time points for Western blotting. (D) The cells were exposed to H_2_O_2_ for 15 min in the presence of 1 µM SERPINA3K or BSA. The phosphorylated PLC-γ1 (p-PLC), ERK1/2 (p-ERK) and Akt (p-AKT) were blotted with phosphorylation-specific antibodies. The total PLC-γ1 (t-PLC), ERK1/2 (t-ERK) and Akt (t-AKT) were blotted with antibodies for total proteins. (E) Protein levels was quantified using densitometry of the Western blotting results (mean±SEM, n = 3). (F) The reduced phosphorylation level of each kinase was shown as percentages of the maximum phosphorylation levels at 15 min exposure (mean±SEM, n = 3). $ P<0.01, under H_2_O_2_ exposure from 1.5 h to 4 h; the U73122 and SERPINA3K-treated cells versus the BSA-treated cells. * P<0.01, versus control cells. # P<0.01, the cells treated with H_2_O_2_, m-3M3FBS and SERPINA3K versus the cells treated with H_2_O_2_ and SERPINA3K. & P<0.01, versus control cells exposed to H_2_O_2_ for 0 min.

To study the mechanism by which H_2_O_2_ induces intracellular calcium overload and necrosis, several specific inhibitors were used to block the signaling pathways known to be activated by H_2_O_2_ and related to calcium overload. Müller-derived rMC-1 cells were treated with 400 µM H_2_O_2_ together with 1 µM SERPINA3K, 250 nM U73122 (PLC inhibitor), 10 µM U0126 (ERK inhibitor) or 100 nM Wortmannin (PI3K inhibitor). BSA and 0.5% DMSO were used as negative control. The cells treated with SERPINA3K and U73122 showed significantly lower intracellular Ca^2+^ concentrations than that in the cells treated with U0126, Wortmannin and BSA, after 1.5 to 4 h incubation (Fig. 6A). Correlated with their effects on Ca^2+^, MTT assay demonstrated that SERPINA3K, U73122 and BAPTA, but not U0126 and Wortmannin, had protective effects on cell viability under the H_2_O_2_ stress (Fig. 6B), suggesting that the reduction of intracellular Ca^2+^ induced by SERPINA3K is not through the ERK or PI3K pathways. Consistently, the protective effect of SERPINA3K, but not that of BAPTA, was offset by 10 µM m-3M3FBS, a specific PLC activator, suggesting that SERPINA3K prevents calcium overload through blocking PLC activation.

### SERPINA3K decreases the H_2_O_2_-induced phosphorylation of PLC-γ1, but not ERK or Akt

The H_2_O_2_ exposure induced the phosphorylation of PLC, ERK and Akt in a time-dependent manner (Fig. 6C). All of these kinases were phosphorylated by H_2_O_2_ as early as 5 min of exposure, and the phosphorylation of the kinases reached peaks at 15 min of exposure to H_2_O_2_ (Fig. 6C, E). At 15 min, the SERPINA3K treatment prevented the phosphorylation of PLC induced by H_2_O_2_ (60% reduction), but did not inhibit the phosphorylation of the ERK or PI3K/AKT kinases, suggesting that SERPINA3K specifically blocks the activation of PLC under oxidative stress (Fig. 6D, F).

### SERPINA3K binds to rMC-1 cells

**Figure 7:**
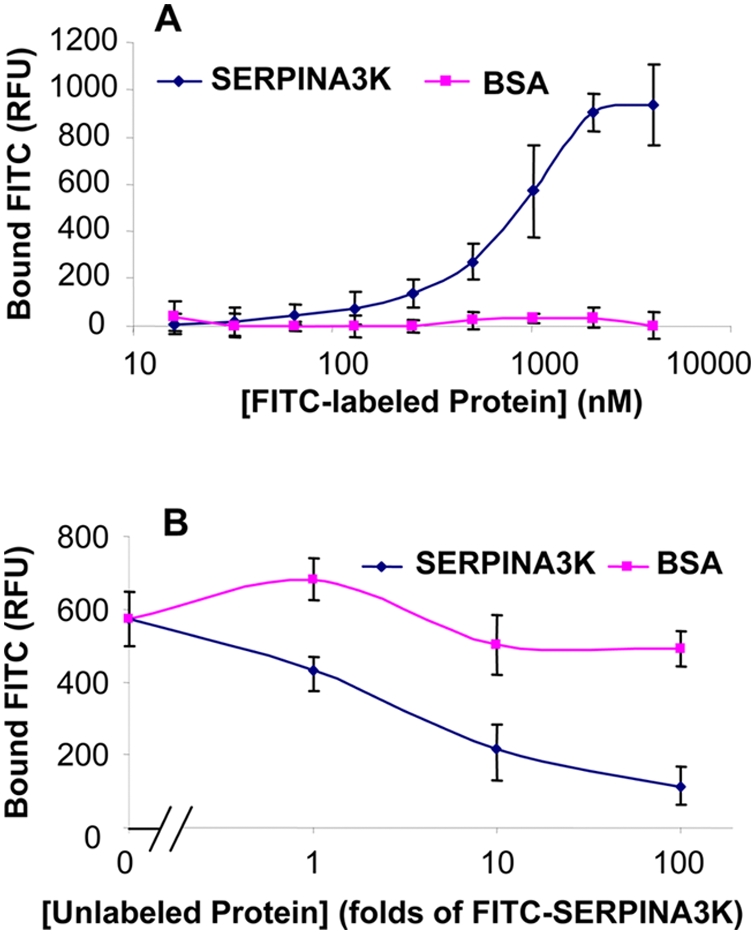
SERPINA3K binds to Müller-derived rMC-1 cells. (A) The rMC-1 cells were incubated with increasing concentrations of FITC-SERPINA3K or FITC-BSA for 1 h. (B) The rMC-1 cells were incubated with 400 nM FITC-SERPINA3K in the presence of different concentrations of unlabeled SERPINA3K or BSA for 1 h. Then the cells were washed three times with PBS. The fluorescence in the cells was measured by a fluorometer using 485/530 nm filter (mean±SEM, n = 8).

To investigate if the protective effect of SERPINA3K is mediated through a receptor on the cell surface, the binding of SERPINA3K on Müller-derived rMC-1 cells was determined. Cultured rMC-1 cells were incubated with increasing concentrations (16–4096 nM) of FITC-labeled SERPINA3K for 1 h followed by 3 washes with PBS. Then the fluorescence intensities on rMC-1 cells were measured with a fluorometer. As shown by Figure 8A, FITC-SERPINA3K displayed a concentration-dependent and saturable binding onto rMC-1 cells with a K_d_ at approximate 700 nM. In contrast, FITC-BSA did not show any significant binding to rMC-1 cells in the same concentration range (Fig. 7A).

To determine the specificity of the binding of SERPINA3K on rMC-1 cells, FITC-SERPINA3K (400 nM) was incubated with the cells in the presence of excess of unlabeled SERPINA3K or BSA (1, 10 or 100 folds over the FITC-SERPINA3K in concentrations). The binding assay showed that the binding of FITC-SERPINA3K to rMC-1 cells was competed off by excess concentrations of unlabeled SERPINA3K, but not by BSA (Fig. 7B), suggesting that the binding of SERPINA3K to Müller-derived rMC-1 cells is reversible and specific.

## Discussion

SERPINA3K is an extracellular serpin. It has been found to function as an anti-angiogenic factor, inhibiting angiogenesis and reducing retinal vascular leakage [11]. The present study revealed a novel function of this serpin, i.e., a protective effect on retinal Müller cells and retinal neuronal cells against oxidative stress-induced damage. Further, our results demonstrate that the protective effect of SERPINA3K is through blocking intracellular calcium overload induced by oxidative stress through inhibition of PLC activation. These results indicate that SERPINA3K is a novel protective factor in the retina and the first extracellular serpin identified which exerts its protective effects through inhibiting PLC activation and blocking intracellular calcium overload.

Müller cells are a type of retinal glial cells and are known to be essential for the structure and function of the retina. The layered arrangements of retinal neurons are maintained by Müller cells [27]. Müller cells produce neurotrophic substances [14] and remove metabolic wastes [15] to support neuronal survival. Müller cell death has been shown to lead to neuron degeneration in a transgenic mouse model [17]. The oxidative damage of Müller cells and retinal neuronal cells has been shown to be an important pathological feature in some ocular diseases, such as diabetic retinopathy or light-induced retinopathy [18], [28], [29]. Our results indicate that SERPINA3K protects Müller cells under the oxidative stress and thus, may have potential neurotrophic function in the retina.

Accumulating evidence shows that retinal neuron degeneration is an important pathological feature contributing to vision impairment in diabetic retinopathy [14]. Our previous studies have shown that SERPINA3K levels are decreased in the retina of STZ-diabetic rats [13]. The present study suggests for the first time that decreased levels of SERPINA3K in diabetes may contribute to retinal neuron degeneration as well as to retinal vascular leakage and retinal neovascularization in diabetic retinopathy.

Oxidative stress is believed to play a major pathogenic role in retinal degeneration as found in diabetic retinopathy and AMD. Recently, antioxidants have been used to ameliorate retinopathy induced by oxidative stress [30], [31]. H_2_O_2_ is a by-product of oxidative stress and can induce a number of cellular responses and cell death. In cultured cells, H_2_O_2_ is commonly used as an oxidative stressor. The H_2_O_2_-induced oxidative stress is known to be affected by multiple factors, such as H_2_O_2_ concentrations, cell types, cell cycle phases and medium components, etc. It has been shown that H_2_O_2_ can activate both the apoptotic pathway [22] and necrotic pathway [23], depending on conditions and cell types. In our model, a short exposure of Müller-derived rMC-1 cells to 400 µM H_2_O_2_ induces necrosis rather than apoptosis, as shown by Annexin-V/PI staining and flow cytometry and DNA fragmentation analyses. The necrotic process is usually mediated through fast cellular responses, such as intracellular calcium overload which subsequently induces protease release to damage the cell. Although another serpin, pigment epithelium-derived factor (PEDF) has also displayed neuroprotective activities, the protective effect of PEDF is through blocking apoptosis of neuronal cells [32]. The present study demonstrated that SERPINA3K blocks calcium overload induced by H_2_O_2_ exposure. This represents a novel mechanism of action for extracellular serpin family members.

To elucidate the mechanism by which SERPINA3K decreases the intracellular calcium overload induced by H_2_O_2_, we have tested three possible mechanisms: blocking intracellular ROS generation induced by H_2_O_2_, directly chelating calcium ions and blocking the intracellular signaling pathway through which H_2_O_2_ stimulates the intracellular calcium increase. Using the CM-H2DCFDA probe, we showed that SERPINA3K did not block the increase of intracellular ROS levels in the cells exposed to H_2_O_2_, although it reduced cell death. Our calcium chelating experiments showed that SERPINA3K did not chelate free calcium ion in a CaCl_2_ solution or in cell lysates. Further, SERPINA3K does not block cell death induced by TBHP which triggers ROS generation but not calcium overload. These results suggest that SERPINA3K is likely to block the signaling pathway between the H_2_O_2_ stress and calcium overload, which is unrelated to its effect on reducing ROS generation.

Multiple intracellular signaling pathways have been shown to regulate intracellular calcium levels. For example, the phosphorylation of NHE-1 by the ERK1/2 MAP kinase pathway has been shown to stimulate the calcium pump [33]. PI3K and Akt are known to promote the voltage-dependent calcium channel [34]. It is well known that PLC is an important regulator of calcium homeostasis via IP3 and DAG [35], and the phosphorylation of PLC-γ1 is an essential step in some calcium regulation pathways [36]. The association of PLC-γ with the H_2_O_2_ stress has been revealed previously, as there is a direct evidence to support the critical role of PLC-γ1 in the H_2_O_2_-induced calcium signaling [37], [38]. To identify the pathway involved in the H_2_O_2_-induced necrosis in rMC-1 cells, specific inhibitors for ERK, PI3K and PLC were used. Among these, only the specific PLC inhibitor blocked the calcium overload and necrosis induced by H_2_O_2_, while blocking the ERK and PI3K pathways did not inhibit calcium overload (Fig. 6). These results suggest that the H_2_O_2_-induced calcium overload is mediated through PLC.

As SERPINA3K is an extracellular serpin, we have investigated if its protective effect is mediated through a receptor. The SERPINA3K binding assay demonstrated a specific and saturable binding of SERPINA3K to Müller-derived rMC-1 cells, suggesting that the SERPINA3K effect may be through a receptor or binding protein on the cell surface. The major SERPINA3K source in the retina is still unclear. However, total levels of SERPINA3K in the retina have been shown to be decreased in diabetic animals [13]. The decreased levels of SERPINA3K in diabetic retinas may contribute to oxidative stress and retinal cell death. In the oxidative stress-induced Müller cell death, the earliest detected event was the H_2_O_2_-induced PLC phosphorylation, at as early as 15 min exposure to H_2_O_2_. The PLC phosphorylation subsequently induced calcium overload which was observed after 2 h exposure to H_2_O_2_, which resulted in an remarkable increase in cell necrosis at 4 h. It is likely that binding of SERPINA3K triggers the activation of an intracellular signaling pathway, leading to the blockade of PLC activation and calcium overload under oxidative stress.

SERPINA3K at the concentrations used showed substantially lower cytotoxicities compared to the calcium chelator (BAPTA) and PLC inhibitor (U73122). At the concentrations used to prevent the H_2_O_2_-induced rMC-1 cell death, both BAPTA and U73122 decreased the cell viability (Fig. S1). At the dose used, SERPINA3K did not induce any detectable decrease in cell viabilities in Müller cells. These observations suggest that SERPINA3K has therapeutic potential for the treatment of some retinal degenerative diseases induced by oxidative stress, such as diabetic retinopathy and light induced retinopathy.

## Materials and Methods

### Cell culture

rMC-1 cells, a cell line derived from rat retinal Müller cells, were a kind gift from Dr. Vijay Sarthy at Northwestern University, and cultured in low glucose (1 g/L) Dulbecco's Modified Eagle's Medium (DMEM) containing 10% fetal bovine serum (FBS, Invitrogen, Carlsbad, CA) [39]. R28 is a cell line derived from rat retinal precursor cells, expressing photoreceptor markers, and is a generous gift from Dr. Gail Seigel at SUNY at Buffalo. Human retinal pigment epithelial (RPE) cell line, hTERT RPE-1, was purchased from ATCC (Manassas, VA). Both of R28 and hTERT RPE-1 cells were cultured in high glucose (4.5 g/L) DMEM containing 10% FBS. Y79 cells, a human retinoblastoma cell line, were purchased from ATCC and cultured in RPMI1640 containing 15% FBS as recommended by ATCC. The cultured cells were exposed to low glucose media containing 1% FBS for 4 h prior to the addition of proteins or compounds, and all of the experiments were performed in the 1% FBS medium. Cultures were always pretreated for 1 hr with SERPINA3K at concentrations indicated for each experiment, to ensure that the protein bound to the cells before the H_2_O_2_ exposure.

### Protein and chemicals

SERPINA3K was cloned into the pET28 vector (Novagen, Madison, WIS) and the construct was transformed into *E. coli* strain BL-21/DE3 (Novagen, Madison, WIS). The expression and purification followed the protocol described previously [11]. Endotoxin test was performed using a limulus amebocyte kit (Biowhittaker, Walkersville, MD).

Bovine serum albumin (BSA) and H_2_O_2_ was purchased from Sigma (St. Louis, MO) and all of the inhibitors were purchased from EMD (San Diego, CA).

### Cell viability analysis

The viable cells were quantified by the MTT (3-(4,5-dimethylthiazol-2-yl)-2.5-diphenyl-2H-tetrazolium bromide, Roche, Indianapolis, IN) cell viability assay following the protocol recommended by the manufacturer. The cultured cells were seeded in 96-well plates (10^4^ cells/well, in 100 µl) and cultured in culture media for 24 h. Following the treatment of SERPINA3K and H_2_O_2_ for desired durations, 10 µl (1 mg/ml) of buffer 1 (MTT) were added to the cells and incubated for 4 h. Then 100 µl of buffer 2 (10% SDS in 0.01M HCl) were added and incubated with the cells for 16 h. The optical absorbance at 570 nm was measured using an ELISA reader.

### Flow cytometry analysis

After the treatment with H_2_O_2_ and SERPINA3K, rMC-1 cells were washed twice with ice-cold PBS and re-suspended in 1× binding buffer (10 mM HEPES, pH 7.4, 140 mM NaCl, 2.5 mM CaCl_2_) at a density of ∼1×10^6^ cells/ml. The cell suspension containing approximate 10^5^ cells was stained with Annexin-V and propidium iodide (PI) (BD, San Jose, CA) for 30 min in the dark. Then 400 µl of 1× binding buffer were added to each sample which was analyzed by flow cytometry to quantify necrotic and apoptotic cells.

### DNA-laddering assay

To test the DNA fragmentation, cells were lysed in buffer 1 (10 mM Tris, 1 mM EDTA and 0.2% Triton X-100) [40]. The cytosolic fraction of the lysates was collected after centrifugation. The supernatant was brought to buffer 2 (150 mM NaCl, 40 mM EDTA, 1% SDS and 200 mg/ml of proteinase K). Fragmented DNA was precipitated with isopropanol and resuspended in DNA loading buffer. The fragmented DNA was resolved by electrophoresis in 2% agarose gels containing ethidium bromide and visualized under the UV light.

### Fluorescent detection of intracellular ROS

To measure intracellular ROS generation, cells were incubated with 10 µM CM-H2DCFDA (Invitrogen, Carlsbad, CA) for 30 min in the dark. After the CM-H2DCFDA entered the cells, the diacetate group was removed by intracellular esterase, trapping the probe inside the cells. Generation of ROS was quantified by measuring the fluorescent oxidation product CM-DCF in the cytosol, at an excitation wavelength of 480 nm and an emission wavelength of 520 nm.

### Measurement of intracellular calcium concentration

To measure the intracellular calcium concentration, rMC-1 cells were incubated with 5 µM Fluo-4/AM (Invitrogen, Carlsbad, CA) at 37°C for 30 min. Then the incubation buffer was replaced by DMEM containing 1% FBS. After the addition of H_2_O_2_ or other reagents, the fluorescence intensity was continuously monitored at an excitation wavelength of 494 nm and an emission wavelength of 516 nm. For the quantification of the free Ca^2+^ concentration *in vitro*, the fluorescence of the Fluo-4/pentapotassium salt (Invitrogen, Carlsbad, CA) was measured. For fluorescent microscopy, the cells were washed three times with PBS prior to capture of the image under a fluorescent microscope.

### SERPINA3K binding assay

SERPINA3K was labeled with FITC using the FITC labeling kit (Invitrogen, Carlsbad, CA) following a protocol recommended by the manufacturer. For the binding assay, rMC-1 cells were seeded in 96-well plates. The cells were incubated with increasing concentrations of FITC-labeled SERPINA3K for 1 h. The unbound FITC-SERPINA3K was removed by 3 washes with PBS. The FITC-SERPINA3K bound to the cells was measured by fluorescence intensity using a fluorometer with an excitation wavelength of 495 nm and an emission wavelength of 520 nm.

### Western blot analysis

One hundred micrograms of total cellular proteins were resolved by SDS-PAGE and then blotted with specific antibodies. Antibodies for PLCγ1 (t-PLC), phosphorylated PLCγ1 at Tyr783 (p-PLC), ERK1/2 (t-ERK), phosphorylated ERK1/2 at Thr202 and Tyr204 (p-ERK), Akt (t-AKT) and phosphorylated Akt at Ser473 (p-AKT) were purchased from Cell Signaling (Danvers, MA) and used at the 1∶1000 dilution.

### Statistical analysis

Student's t test was used in all statistical analyses, and statistical significance was accepted when the P value was less than 0.05.

## Supporting Information

Figure S1 SERPINA3K has no cytotoxicity in Müller-derived rMC-1 cells. The rMC-1 cells were treated with 1 µM SERPINA3K, 10 µM BAPTA or 250 nM U73122 for 24 h. The same concentration of BSA and DMSO were used as controls. The cell viability was measured with the MTT assay (mean±SEM, n = 3). At the concentrations used, SERPINA3K did not decrease cell viability while BAPTA and U73122 showed significant decrease of viable cells. * P<0.05, the cells treated with BAPTA or U73122 versus control cells.
(0.23 MB TIF)

## References

[1] Gettins PG (2002). Serpin structure, mechanism, and function.. Chem Rev.

[2] Silverman GA, Bird PI, Carrell RW, Church FC, Coughlin PB (2001). The serpins are an expanding superfamily of structurally similar but functionally diverse proteins. Evolution, mechanism of inhibition, novel functions, and a revised nomenclature.. J Biol Chem.

[3] Luke CJ, Pak SC, Askew YS, Naviglia TL, Askew DJ (2007). An intracellular serpin regulates necrosis by inhibiting the induction and sequelae of lysosomal injury.. Cell.

[4] Abraham MC, Shaham S (2007). Necrosis and the serpin under't.. Dev Cell.

[5] Chao J, Tillman DM, Wang MY, Margolius HS, Chao L (1986). Identification of a new tissue-kallikrein-binding protein.. Biochem J.

[6] Chao J, Chai KX, Chen LM, Xiong W, Chao S (1990). Tissue kallikrein-binding protein is a serpin. I. Purification, characterization, and distribution in normotensive and spontaneously hypertensive rats.. J Biol Chem.

[7] Clements JA (1989). The glandular kallikrein family of enzymes: tissue-specific expression and hormonal regulation.. Endocr Rev.

[8] Murray SR, Chao J, Lin FK, Chao L (1990). Kallikrein multigene families and the regulation of their expression.. J Cardiovasc Pharmacol.

[9] Bhoola KD, Figueroa CD, Worthy K (1992). Bioregulation of kinins: kallikreins, kininogens, and kininases.. Pharmacol Rev.

[10] Ma JX, Yang Z, Chao J, Chao L (1995). Intramuscular delivery of rat kallikrein-binding protein gene reverses hypotension in transgenic mice expressing human tissue kallikrein.. J Biol Chem.

[11] Gao G, Shao C, Zhang SX, Dudley A, Fant J (2003). Kallikrein-binding protein inhibits retinal neovascularization and decreases vascular leakage.. Diabetologia.

[12] Miao RQ, Agata J, Chao L, Chao J (2002). Kallistatin is a new inhibitor of angiogenesis and tumor growth.. Blood.

[13] Hatcher HC, Ma JX, Chao J, Chao L, Ottlecz A (1997). Kallikrein-binding protein levels are reduced in the retinas of streptozotocin-induced diabetic rats.. Invest Ophthalmol Vis Sci.

[14] Newman E, Reichenbach A (1996). The Muller cell: a functional element of the retina.. Trends Neurosci.

[15] Poitry S, Poitry-Yamate C, Ueberfeld J, MacLeish PR, Tsacopoulos M (2000). Mechanisms of glutamate metabolic signaling in retinal glial (Muller) cells.. J Neurosci.

[16] Newman EA (1993). Inward-rectifying potassium channels in retinal glial (Muller) cells.. J Neurosci Res.

[17] Dubois-Dauphin M, Poitry-Yamate C, de Bilbao F, Julliard AK, Jourdan F (2000). Early postnatal Muller cell death leads to retinal but not optic nerve degeneration in NSE-Hu-Bcl-2 transgenic mice.. Neuroscience.

[18] Puro DG (2002). Diabetes-induced dysfunction of retinal Muller cells.. Trans Am Ophthalmol Soc.

[19] Altomare E, Grattagliano I, Vendemaile G, Micelli-Ferrari T, Signorile A (1997). Oxidative protein damage in human diabetic eye: evidence of a retinal participation.. Eur J Clin Invest.

[20] D'Autreaux B, Toledano MB (2007). ROS as signalling molecules: mechanisms that generate specificity in ROS homeostasis.. Nat Rev Mol Cell Biol.

[21] Kowluru RA, Chan PS (2007). Oxidative stress and diabetic retinopathy.. Exp Diabetes Res.

[22] Clement MV, Ponton A, Pervaiz S (1998). Apoptosis induced by hydrogen peroxide is mediated by decreased superoxide anion concentration and reduction of intracellular milieu.. FEBS Lett.

[23] Wang X, Ryter SW, Dai C, Tang ZL, Watkins SC (2003). Necrotic cell death in response to oxidant stress involves the activation of the apoptogenic caspase-8/bid pathway.. J Biol Chem.

[24] Halliwell B (1992). Reactive oxygen species and the central nervous system.. J Neurochem.

[25] Syntichaki P, Tavernarakis N (2002). Death by necrosis. Uncontrollable catastrophe, or is there order behind the chaos?. EMBO Rep.

[26] Zong WX, Thompson CB (2006). Necrotic death as a cell fate.. Genes Dev.

[27] Willbold E, Berger J, Reinicke M, Wolburg H (1997). On the role of Muller glia cells in histogenesis: only retinal spheroids, but not tectal, telencephalic and cerebellar spheroids develop histotypical patterns.. J Hirnforsch.

[28] Anderson RE, Kretzer FL, Rapp LM (1994). Free radicals and ocular disease.. Adv Exp Med Biol.

[29] Ohira A, Tanito M, Kaidzu S, Kondo T (2003). Glutathione peroxidase induced in rat retinas to counteract photic injury.. Invest Ophthalmol Vis Sci.

[30] Kowluru RA, Engerman RL, Case GL, Kern TS (2001). Retinal glutamate in diabetes and effect of antioxidants.. Neurochem Int.

[31] Gosbell AD, Stefanovic N, Scurr LL, Pete J, Kola I (2006). Retinal light damage: structural and functional effects of the antioxidant glutathione peroxidase-1.. Invest Ophthalmol Vis Sci.

[32] Cao W, Tombran-Tink J, Chen W, Mrazek D, Elias R (1999). Pigment epithelium-derived factor protects cultured retinal neurons against hydrogen peroxide-induced cell death.. J Neurosci Res.

[33] Rothstein EC, Byron KL, Reed RE, Fliegel L, Lucchesi PA (2002). H(2)O(2)-induced Ca(2+) overload in NRVM involves ERK1/2 MAP kinases: role for an NHE-1-dependent pathway.. Am J Physiol Heart Circ Physiol.

[34] Viard P, Butcher AJ, Halet G, Davies A, Nurnberg B (2004). PI3K promotes voltage-dependent calcium channel trafficking to the plasma membrane.. Nat Neurosci.

[35] Muallem S (1989). Calcium transport pathways of pancreatic acinar cells.. Annu Rev Physiol.

[36] Tkaczyk C, Beaven MA, Brachman SM, Metcalfe DD, Gilfillan AM (2003). The phospholipase C gamma 1-dependent pathway of Fc epsilon RI-mediated mast cell activation is regulated independently of phosphatidylinositol 3-kinase.. J Biol Chem.

[37] Hong JH, Moon SJ, Byun HM, Kim MS, Jo H (2006). Critical role of phospholipase Cgamma1 in the generation of H2O2-evoked [Ca2+]i oscillations in cultured rat cortical astrocytes.. J Biol Chem.

[38] Rhee SG, Bae YS (1997). Regulation of phosphoinositide-specific phospholipase C isozymes.. J Biol Chem.

[39] Sarthy VP, Brodjian SJ, Dutt K, Kennedy BN, French RP (1998). Establishment and characterization of a retinal Muller cell line.. Invest Ophthalmol Vis Sci.

[40] Canelles M, Delgado MD, Hyland KM, Lerga A, Richard C (1997). Max and inhibitory c-Myc mutants induce erythroid differentiation and resistance to apoptosis in human myeloid leukemia cells.. Oncogene.

