# Supplementary figures and images for: SERPINA3K Prevents Oxidative Stress Induced Necrotic Cell Death by Inhibiting Calcium Overload
Source: PLoS One. 2008 Dec 30;3(12):e4077. doi: 10.1371/journal.pone.0004077 (PMC2605247; doi:10.1371/journal.pone.0004077)

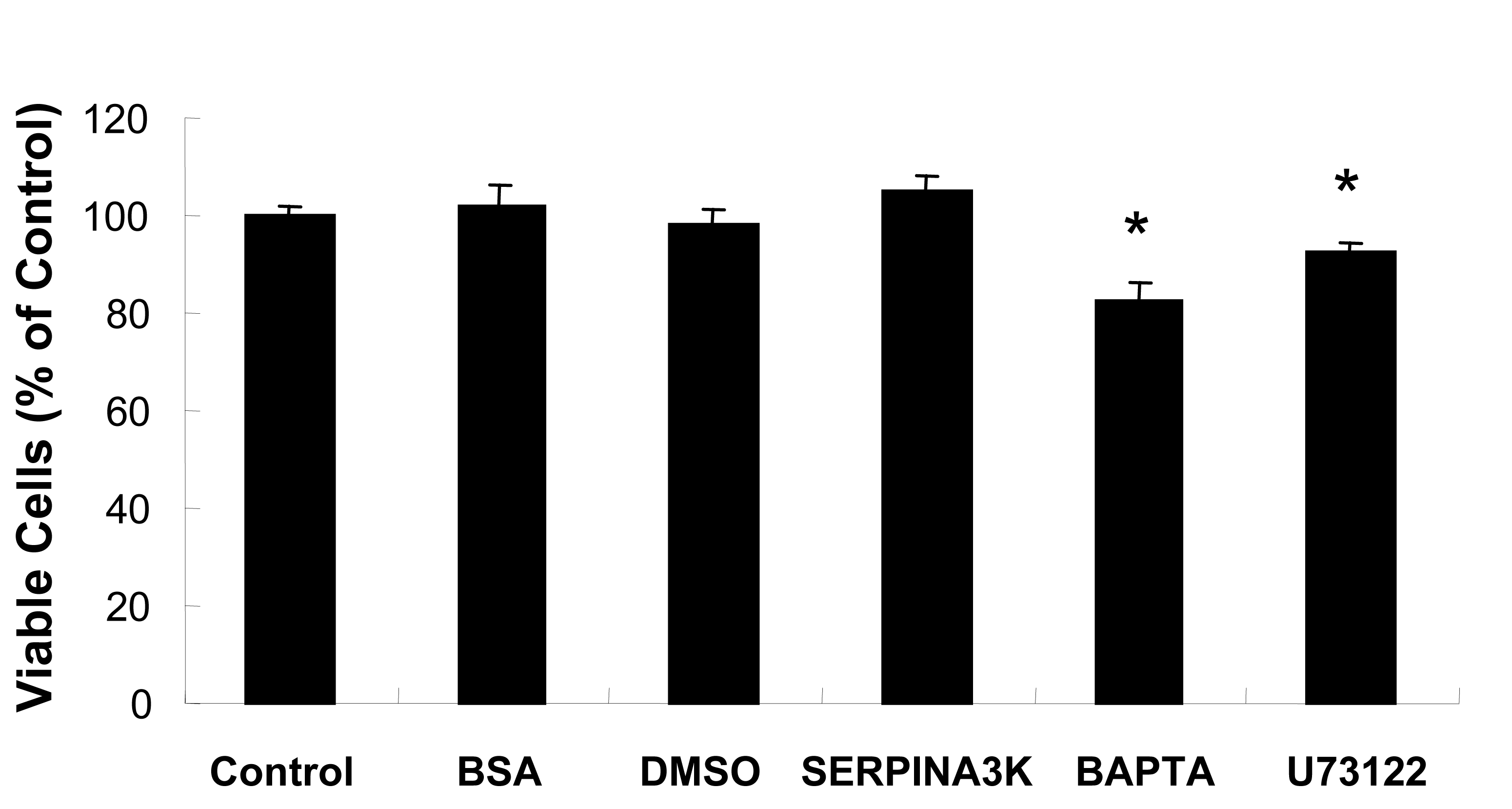

Supplement: Figure S1 — SERPINA3K has no cytotoxicity in Müller-derived rMC-1 cells. The rMC-1 cells were treated with 1 µM SERPINA3K, 10 µM BAPTA or 250 nM U73122 for 24 h. The same concentration of BSA and DMSO were used as controls. The cell viability was measured with the MTT assay (mean±SEM, n = 3). At the concentrations used, SERPINA3K did not decrease cell viability while BAPTA and U73122 showed significant decrease of viable cells. * P<0.05, the cells treated with BAPTA or U73122 versus control cells. (0.23 MB TIF) [file pone.0004077.s001.tif]
